# Lethal multiple colon necrosis and perforation due to fulminant amoebic colitis: a surgical case report and literature review

**DOI:** 10.1186/s40792-020-01095-2

**Published:** 2021-01-20

**Authors:** Takahiro Tomino, Mizuki Ninomiya, Ryosuke Minagawa, Rumi Matono, Yumi Oshiro, Daichi Kitahara, Takuma Izumi, Daisuke Taniguchi, Kosuke Hirose, Yuichiro Kajiwara, Kazuhito Minami, Takashi Nishizaki

**Affiliations:** 1 Department of Surgery, Matsuyama Red Cross Hospital, 1, Bunkyo-cho, Matsuyama-shi, Ehime, 790-8524 Japan; 2 Department of Diagnostic Pathology, Matsuyama Red Cross Hospital, Ehime, Japan; 3 Department of Diagnostic Pathology, Fukuoka Red Cross Hospital, Fukuoka, Japan

**Keywords:** Fulminant amoebic colitis, Bowel perforation, Intestinal necrosis, Colectomy, Serological testing, Metronidazole

## Abstract

**Background:**

Amoebiasis caused by the protozoan species *Entamoeba histolytica* rarely develops into fulminant amoebic colitis (FAC), but when it does, it shows an aggressive clinical course including colonic perforation, necrotizing colitis, and high mortality. Surgical treatment for FAC patients should be carried out urgently. However, even after surgery, the mortality rate can be 40–50%. Although FAC is one of the most unfavorable surgical diseases with a poor prognosis, there are a few reports on the perioperative diagnosis and management of FAC based on autopsy findings. We herein report the surgical case of a 64-year-old man who developed multiple colon necrosis and perforation due to FAC. A detailed autopsy revealed FAC as the cause of death. Additionally, we reviewed the existing literature on FAC patients who underwent surgery and followed their perioperative diagnosis and management.

**Case presentation:**

A 64-year-old man presented with anorexia, diarrhea, and altered consciousness on arrival to our hospital. Computed tomography revealed a large mass in the upper right lobe of his lung, and the patient was admitted for close investigation. Bloody diarrhea, lower abdominal pain, and hypotension were observed soon after admission. Urgent abdominal contrast-enhanced computed tomography scan revealed extensive intestinal ischemia, intestinal pneumatosis, and free intra-abdominal gas. The preoperative diagnosis was bowel necrosis and perforation with intussusception of the small intestinal tumor. Emergency subtotal colectomy and enterectomy were performed soon after the contrast-enhanced computed tomography. He was taken to an intensive care unit after surgery. However, he could not recover from sepsis and died with disseminated intravascular coagulation and multiple organ failure on the 10th-day post-surgery. A histopathological examination of the resected colon showed transmural necrosis and massive amoebae invasion. He was diagnosed with FAC. An autopsy revealed that he had developed pulmonary large cell carcinoma with small intestinal metastasis. The death was caused by intestinal ischemia, necrosis and the perforation of the residual bowel caused by amoebae invasion.

**Conclusions:**

Since FAC is a lethal disease with a high mortality rate and antibiotic therapies except metronidazole are ineffective, preoperative serological testing and perioperative metronidazole therapy in FAC patients can dramatically improve their survival rates.

## Background

Amoebiasis is a parasitic infection caused by the protozoan species *Entamoeba histolytica*. A majority of infected patients remain asymptomatic. However, in some cases of *E. histolytica* infection, the infected patients develop amoebic colitis, defined as amoebic diarrhea with a discharge of mucus or blood, which occurs when amoeba breach the mucosal barrier and travel through the portal circulation to the liver, where they can cause liver abscesses [1]. Fulminant amoebic colitis (FAC), which presents with a more aggressive clinical course including colonic perforation and necrotizing colitis, is a rare condition with a high mortality rate (> 55%) [2]. Surgical treatment for FAC patients should be carried out urgently. Even after surgical treatment, the mortality rate is reported to be 40–50% [3, 4]. Although FAC is one of the most unfavorable surgical diseases with a poor prognosis, there are a few reports on the perioperative diagnosis and management of FAC based on autopsy findings. We herein report the surgical case of a 64-year-old man who developed multiple colon necrosis and perforation due to FAC. Massive amoebae invasion was recognized with a postoperative histopathological examination. Moreover, a detailed autopsy identified intestinal ischemia, necrosis and the perforation of the residual bowel caused by amoebae invasion as the cause of death. We also included a review of the literature on FAC patients undergoing surgery and analyzed their perioperative diagnosis and management.

## Case presentation

**Fig. 1:**
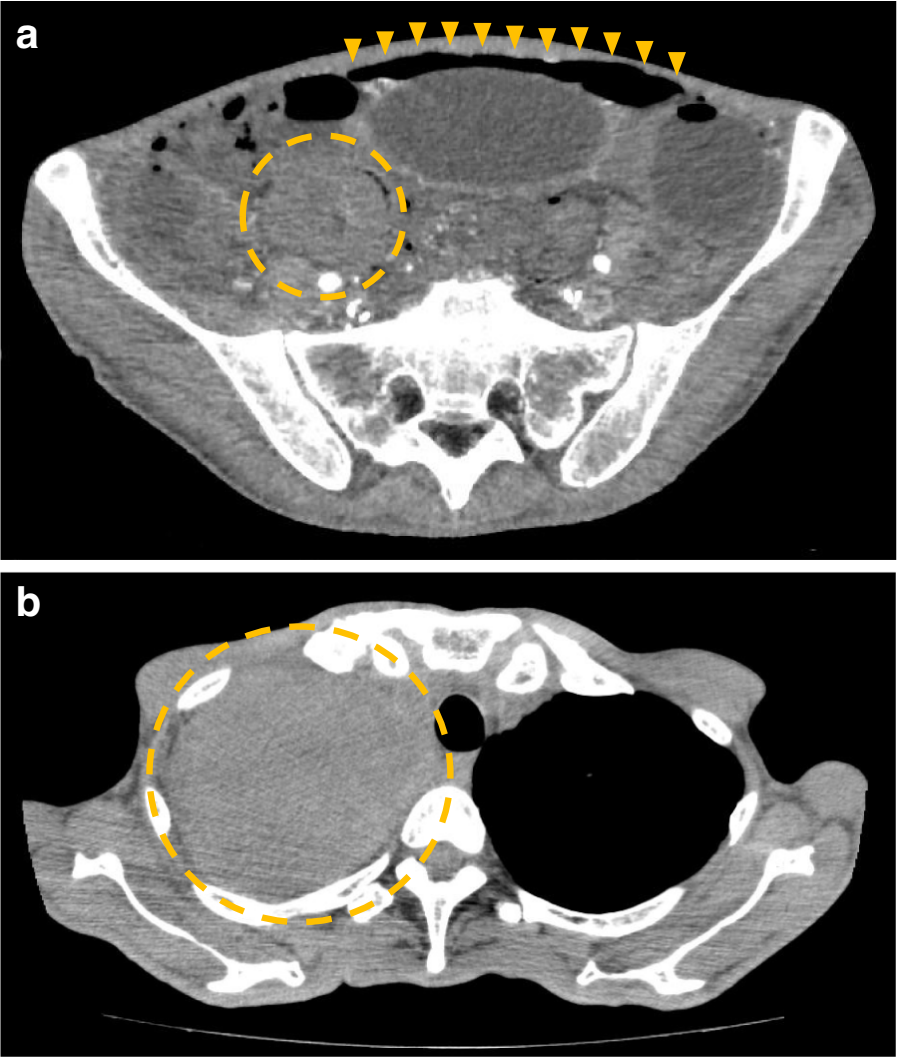
Computed tomography findings. **a** Intestinal ischemia was noted in almost all intestinal tracts. Extensive free intra-abdominal gas was noted in the upper and lower abdomen (arrowheads). Intestinal pneumatosis and intussusception of the small intestinal tumor were noted in the right lower abdomen (circle). **b** A large mass was found in the upper right lobe of patient’s lung (circle)

**Fig. 2:**
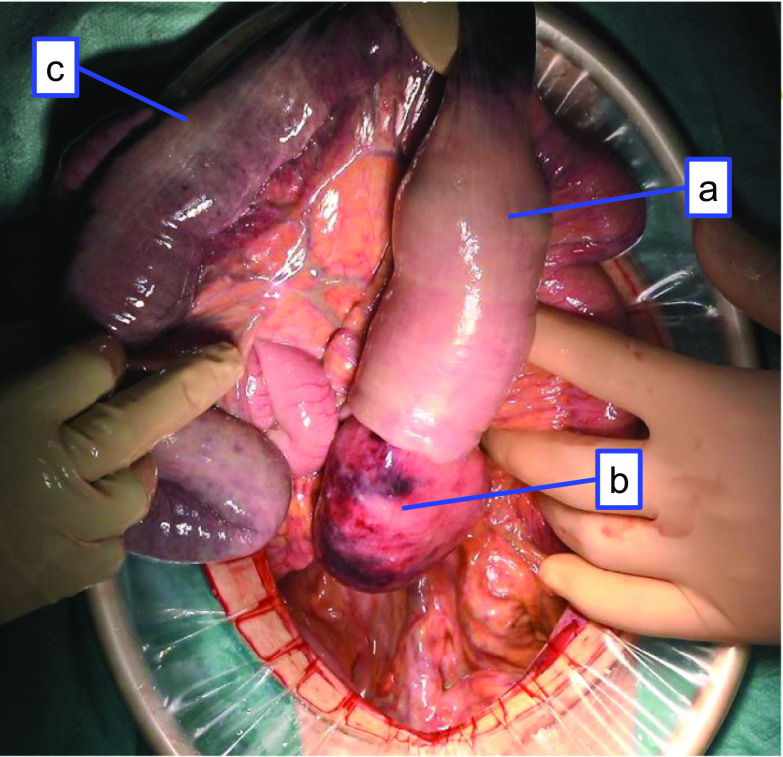
Intraoperative findings. **a** A jejunal tumor telescoped inside of the oral jejunum which caused intussusception. **b** The jejunum had necrotic or ischemic change. **c** The transverse colon had necrotic or ischemic change

A 64-year-old man presenting with anorexia, diarrhea, and altered consciousness was brought to our hospital. Computed tomography (CT) revealed a large mass in the upper right lobe of his lung and the patient was admitted for a close investigation into the lung mass on the same day. Bloody diarrhea, lower abdominal pain, and hypotension were observed soon after admission, and he was referred to us. The patient had a past medical history of hypertension and depression and a family history of lung and laryngeal cancer. He reported a history of smoking 20 cigarettes/day for 44 years, but had no history of consuming alcohol. His physical examination was unremarkable except for mild tenderness in the lower abdomen and severe emaciation. Laboratory results showed significant anemia, leukocytosis, renal failure, and a coagulation disorder with a white blood cell count of 15.4 × 10^3^/μL (86.5% neutrophils), hemoglobin level of 7.2 g/dL, blood urea nitrogen level of 69.4 mg/dL, serum creatinine level of 1.9 mg/dL, prothrombin time 27.4%, and an activated partial thromboplastin time of 46.1 s. The result of his HIV-antibody test was negative. Urgent abdominal contrast-enhanced CT scan was performed due to a sudden change in patient’s condition. It revealed extensive intestinal ischemia, intestinal pneumatosis, free intra-abdominal gas, intussusception of a small intestinal tumor, and a small amount of ascites (Fig. 1a). Chest CT also revealed a large mass in his right upper lobe of the lung (Fig. 1b). The preoperative diagnosis was bowel necrosis and perforation with intussusception of small intestinal tumor. The pulmonary tumor was considered not to be directly associated with his abdominal presentation. Accordingly, emergency subtotal colectomy and enterectomy were performed soon after the contrast-enhanced CT. A jejunal tumor was found telescoped inside of the oral jejunum which caused intussusception (Fig. 2a). We resected the following intestinal tracts which had necrotic or ischemic change: the jejunum (Fig. 2b), part of the ileum, the transverse (Fig. 2c), descending, and sigmoid colon, and the upper rectum. The residual intestine had no necrotic, ischemic, or perforation change. The residual jejunum and ileum were anastomosed with an automatic suture device. Exteriorization of the ascending colon was performed at the end of surgery. After surgery, he was taken to the intensive care unit and placed on a ventilator with an antibiotic treatment including meropenem for 10 days. Additionally, vasopressors including noradrenaline and adrenaline were administered to keep his blood pressure above 80 mmHg. Candida was positive in culture test of abdominal drainage fluid on the 5th-day post-surgery and micafungin treatment was soon started. In his postoperative course, he experienced septic shock and despite our intensive care, the patient did not recover from sepsis and died with disseminated intravascular coagulation and multiple organ failure 10 days after surgery.

**Fig. 3:**
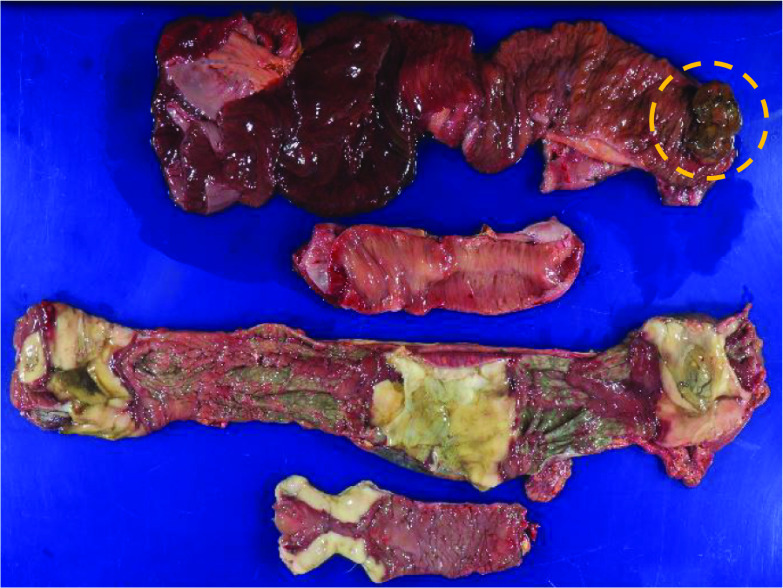
Findings in the resected specimen. In this figure, the first and second intestinal tracts, from the top, correspond to the resected jejunum and part of the ileum. The small intestinal tumor was found in the oral side of the resected jejunum (circle). The third intestinal tract from the top represents the resected transverse colon and descending colon. The fourth intestinal tract from the top is the resected sigmoid colon. The dark-red colored segments of resected specimens showed ischemic changes. Particularly, the resected white colored segment of colon had multiple necroses with ulceration

**Fig. 4:**
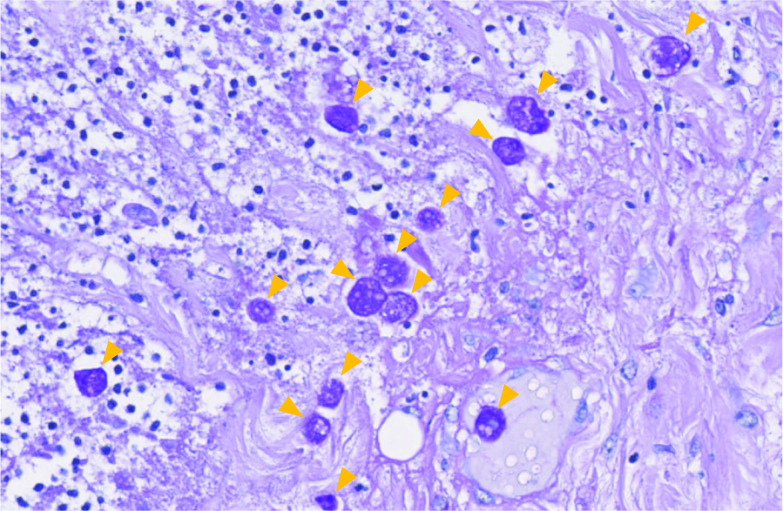
Histopathological findings in the resected colon. Massive amoebae invasion was observed using periodic acid–Schiff stains

**Fig. 5:**
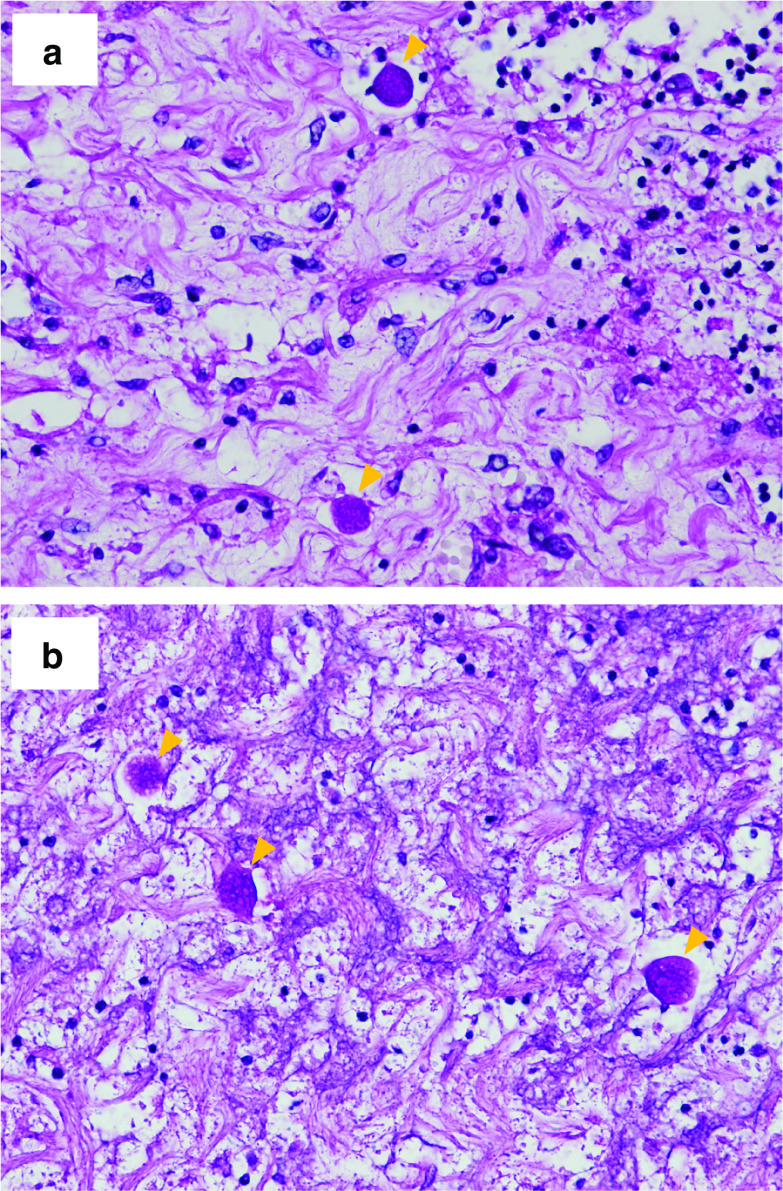
Autopsy findings in the residual bowel. Amoebae invasion was observed in the residual ileum (**a**) and rectum (**b**) using periodic acid–Schiff stains

The resected specimens showed multiple extensive necrotic or ischemic areas. Particularly, the resected transverse, descending, and sigmoid colon areas had multiple sites of necrosis with ulceration. A mass-type tumor was observed in the resected jejunum (Fig. 3). On histopathological examination of the resected specimens with periodic acid–Schiff stains, the dark-red colored segments of the bowel showed ischemic changes such as epithelial desquamation, bleeding, and congestion while the white colored segments of the colon showed transmural necrosis and massive amoebae invasion (Fig. 4). He was diagnosed with FAC based on the histopathological findings of the resected specimens. The mass-type tumor in the resected jejunum consisted of markedly atypical and polymorphic cells and was poorly differentiated. Therefore, it was difficult to define whether it was a primary or metastatic tumor. We performed an autopsy to determine the cause of his death after obtaining the consent of the patient's family. On pathoanatomical examination, extensive transmural ischemic and necrotic areas, as well as multiple perforations, were observed in the residual ileum and rectum. Surprisingly, the amoebic invasion was also observed in the residual ileum (Fig. 5a) and rectum (Fig. 5b). The celiac, superior mesenteric, and inferior mesenteric artery showed no thrombus or tumor embolus. The histological determination of the large mass in the upper right lobe of the patient’s lung was large cell carcinoma, which was similar to the jejunum tumor. In conclusion, the autopsy revealed that he had developed pulmonary large cell carcinoma with small intestinal metastasis and the cause of his death was intestinal ischemia, necrosis and the perforation of the residual ileum and rectum caused by amoebae invasion.

## Discussion

We conducted a systematic review of the literature using the PubMed search engine and found 25 surgical cases of FAC. These 25 cases, along with our case report, are summarized in Table 1 [5–29].

**Table 1:** The present case and 25 reported surgical cases of FAC [5–29]

References	Age	Gender	Preoperative stool test	Preoperative serological test	Preoperative endoscopy	Preoperative diagnosis	Definitive diagnosis examination	Operative procedure	Usage of metronidazole	Outcome
Essenhigh [5]	56	M	No	No	No	Diverticular perforation	Postoperative pathological examination	Subtotal colectomy	No	Dead
Greenstein [6]	36	M	Yes	Yes	No	Amoebic colitis	Stool test, serological test	Ileostomy, colostomy	Yes	Alive
Babb [7]	55	F	No	No	No	Sepsis, acute abdomen	Postoperative pathological examination	Total colectomy	Yes	Alive
Park [8]	49	M	No	No	No	Diverticular perforation	Postoperative pathological examination	Subtotal colectomy	No	Dead
Rennert [9]	0	M	Yes	No	Yes	Necrotizing enterocolitis	Postoperative pathological examination	Resection of rectosigmoid	No	Dead
Shimada [10]	62	M	No	No	Yes	Perforating appendicitis with localized peritonitis → panperitonitis	Postoperative pathological examination	Total colectomy	Yes	Dead
Ishida [11]	48	M	No	No	Yes	Amoebic colitis	Biopsy	Total colectomy	Yes	Alive
Ng [12]	57	M	No	No	No	Obstructing right-sided colonic carcinoma, with liver metastases	Postoperative pathological examination	Right hemicolectomy	Yes	Alive
McGregor [13]	58	F	No	No	No	Bowel perforation	Postoperative pathological examination	Subtotal colectomy	Yes	Alive
Gupta [14]	68	M	No	No	No	Not listed	Postoperative pathological examination	Total colectomy	Yes	Dead
Hanaoka [15]	52	M	No	No	Yes	Amoebic colitis	Biopsy	Colostomy	Yes	Alive
Khan [16]	45	M	No	Yes	Yes	Amoebic colitis and liver abscess	Serological test	Right hemicolectomy	Yes	Alive
Koh [17]	58	M	No	No	Yes	Amoebic colitis	Biopsy	Total colectomy	Yes	Dead
Ishioka [18]	39	M	Yes	No	No	Bowel perforation	Postoperative pathological examination, postoperative serological test	Right hemicolectomy	Yes	Alive
Arora [19]	54	M	No	Yes	No	Amoebic colitis and liver abscess	Serological test	Not listed	Yes	Dead
Lee [20]	47	F	Yes	No	Yes	Intestinal vasculitis	Postoperative pathological examination	Total colectomy	Yes	Alive
Forteza [21]	33	F	No	No	No	Steroid induced colitis with cecal perforation	Postoperative pathological examination	Right hemicolectomy	Yes	Alive
Pirti [22]	62	M	No	No	No	Ileus	Postoperative pathological examination	Right hemicolectomy	Yes	Alive
Saha [23]	65	M	No	No	No	Perforative peritonitis in an obstructing right-sided colonic carcinoma	Postoperative pathological examination	Resection of cecal	Not listed	Not listed
Raj [24]	4	M	No	No	No	Bowel perforation	Postoperative biopsy	Ascending colostomy	Yes	Alive
Goto [25]	30	F	No	No	No	Bowel perforation	Postoperative pathological examination	Subtotal colectomy	Yes	Alive
Guzmán [26]	70	F	Yes	No	No	Amoebic colitis	Stool test	Total colectomy	Not listed	Dead
Chandnani [27]	39	M	Yes	Yes	Yes	Amoebic colitis	Serological test	Right hemicolectomy	Yes	Alive
Wingfield [28]	56	M	Yes	No	No	Severe pancolitis with perforations of the cecum and sigmoid colon	Postoperative pathological examination	Subtotal colectomy	Yes	Alive
Wang [29]	49	M	No	No	Yes	Bowel perforation	Postoperative pathological examination	Total colectomy	Not listed	Dead
Present case	64	M	No	No	No	Bowel perforation and intussusception of small intestine tumor	Postoperative pathological examination	Subtotal colectomy and enterectomy	No	Dead

*FAC* fulminant amoebic colitis, *M* male, *F* female

Taken together, we could observe that the median patient age was 53 years (range 0–70 years) and most patients were men (77%) (Table 1). The preoperative diagnosis of FAC is very difficult and consequently, in 18 out of 26 cases (69%) there was no preoperative diagnosis of FAC. The diagnosis of FAC, in these cases, was determined postoperatively based on the pathological examination of extracted specimens or a postoperative endoscopic biopsy (Table 1). The clinical symptoms of amoebic colitis can range from mild diarrhea, abdominal cramps, and right-lower quadrant tenderness, to severe abdominal cramps, fever, and mucoid or bloody diarrhea [30]. The differential diagnosis of a diarrheal illness with bloody stool also includes a probable infection by *Shigella*, *Salmonella*, *Campylobacter* species and enteroinvasive and enterohemorrhagic *Escherichia coli*. Non-infectious causes include inflammatory bowel disease, ischemic colitis, diverticulitis, and arteriovenous malformation [31]. Therefore, deriving a preoperative diagnosis of amoebic colitis from clinical symptoms is difficult. Although several antigenic and molecular diagnostic tools have been developed over the years, the most commonly used methods for the diagnosis of intestinal amoebiasis are stool test or intestinal biopsy by microscopy [32]. However, despite these developments, the diagnosis of amoebic colitis remains problematic. The reported sensitivity of microscopic stool test for identifying amoebic protozoa ranges from 25–60% [31]; on the other hand, the characteristic endoscopic findings of amoebic colitis (discrete ulcerations or erosions with white or yellow exudates) may mimic other forms of colonic disease, such as Crohn’s colitis [33–35]. Some patients with acute colitis, especially where amoebiasis is suspected on clinical grounds, will benefit from colonoscopy or flexible sigmoidoscopy with an examination of scrapings and biopsy samples for amoebic trophozoites [36]. In the case of FAC, peritonitis and gastrointestinal perforation are clinically suspected, so endoscopy is often avoided. This dilemma makes the preoperative diagnosis of FAC even more difficult than that of amoebic colitis. Discrete ulcers or erosions with exudates were recognized in the cecum of 93% and in the rectum of 45% of patients with amoebic colitis [33]. More than 50% of FAC cases are associated with coexisting amoebic liver abscess [16]. In our literature review, we found that all cases with liver abscess could diagnose amoebic colitis (Table 1). Moreover, pregnant women, immunocompromised individuals, and patients receiving corticosteroids are especially at risk of fulminant disease, and associations with diabetes and alcohol use have also been reported. [37, 38]. The location of necrotizing enteritis, coexisting liver abscess, past medical history, and social history may be helpful for the preoperative diagnosis of FAC. We found very few reports of FAC associated with cancer. Hanaoka, et al. have been reported of FAC during chemotherapy for advanced gastric cancer [15]. There were no reports of FAC associated with lung cancer. In this review, 2 of 7 patients (29%) who received stool tests and 3 of 9 patients (33%) who underwent endoscopy reached a definite diagnosis of FAC. All patients who received serological testing also reached a definite FAC diagnosis. Furthermore, 5 of 8 patients (63%) who reached the definitive diagnosis of FAC preoperatively survived after surgery (Table 1). Serological testing with high accuracy is essential to make a preoperative diagnosis of FAC.

Nitroimidazoles, particularly metronidazole, are the mainstay of therapy for invasive amoebiasis. Approximately, 90% of patients who present with mild-to-moderate amoebic dysentery show a response to nitroimidazole therapy. In the case of FAC, it is prudent to add broad-spectrum antibiotics to treat intestinal bacteria that may spill into the peritoneum [31]. Furthermore, all patients who did not receive metronidazole therapy died after surgery; in contrast, 15 out of 19 patients (79%) who received metronidazole recovered after surgery (Table 1). The use of metronidazole in perioperative FAC patients can dramatically improve their mortality rates. The mortality of FAC patients who received subtotal or total colectomy was reported to be 57% (8 of 14 cases) (Table 1). This suggests that patients with severe FAC who require aggressive resection have a poor prognosis. Even in those patients, the perioperative use of metronidazole markedly improved their survival rates. In the reports, describing whether metronidazole therapy was used or not, of severe FAC patients requiring aggressive resection, all patients who did not receive metronidazole therapy died after surgery (Table 1). In contrast, five out of nine patients (56%) who received metronidazole therapy recovered after surgery (Table 1). In short, for patients with severe FAC, requiring aggressive resection, who did not receive metronidazole therapy had a far worse prognosis than that of patients who received it. In the present case, the administration of metronidazole might have changed the patient’s clinical course. Due to the high mortality associated with FAC and the effectiveness of metronidazole therapy, patients with clinically suspected FAC based on the location of necrotizing enteritis, coexisting liver abscess, and case history should receive metronidazole therapy immediately. Considering our autopsy findings that showed residual intestinal tract after surgery was infected with amoeba, it is necessary to control the amoebic infection of the residual intestinal tract after the resection of the necrotic or ischemic intestinal tract. The persistence of amoeba infection after surgery is considered to be one of the reasons for poor prognosis in surgical cases of FAC.

## Conclusion

To summarize, FAC is one of the lethal diseases with a high mortality rate. Antibiotic therapies except metronidazole are ineffective. Therefore, preoperative serological testing and perioperative metronidazole therapy in FAC patients can dramatically improve their survival rates. Further studies to track and evaluate FAC cases are warranted to comprehensively understand the etiology of FAC.

## Data Availability

The datasets used and/or analyzed during the current study are available from the corresponding author on reasonable request.

## Abbreviations

FAC: Fulminant amoebic colitis
CT: Computed tomography
